# Defining Recommendations to Guide User Interface Design: Multimethod Approach

**DOI:** 10.2196/37894

**Published:** 2022-09-30

**Authors:** Ceci Diehl, Ana Martins, Ana Almeida, Telmo Silva, Óscar Ribeiro, Gonçalo Santinha, Nelson Rocha, Anabela G Silva

**Affiliations:** 1 Digital Media and Interaction Research Centre (DigiMedia) Department of Communication and Art University of Aveiro Aveiro Portugal; 2 Center for Health Technology and Services Research University of Aveiro Aveiro Portugal; 3 Center for Health Technology and Services Research School of Health Sciences University of Aveiro Aveiro Portugal; 4 Center for Health Technology and Services Research Department of Education and Psychology University of Aveiro Aveiro Portugal; 5 Governance, Competitiveness and Public Policies Department of Social, Political and Territorial Sciences University of Aveiro Aveiro Portugal; 6 Institute of Electronics and Informatics Engineering of Aveiro Department of Medical Sciences University of Aveiro Aveiro Portugal

**Keywords:** user interface design, usability principles, interaction paradigm, generic recommendations, specific recommendations

## Abstract

**Background:**

For the development of digital solutions, different aspects of user interface design must be taken into consideration. Different technologies, interaction paradigms, user characteristics and needs, and interface design components are some of the aspects that designers and developers should pay attention to when designing a solution. Many user interface design recommendations for different digital solutions and user profiles are found in the literature, but these recommendations have numerous similarities, contradictions, and different levels of detail. A detailed critical analysis is needed that compares, evaluates, and validates existing recommendations and allows the definition of a practical set of recommendations.

**Objective:**

This study aimed to analyze and synthesize existing user interface design recommendations and propose a practical set of recommendations that guide the development of different technologies.

**Methods:**

Based on previous studies, a set of recommendations on user interface design was generated following 4 steps: (1) interview with user interface design experts; (2) analysis of the experts’ feedback and drafting of a set of recommendations; (3) reanalysis of the shorter list of recommendations by a group of experts; and (4) refining and finalizing the list.

**Results:**

The findings allowed us to define a set of 174 recommendations divided into 12 categories, according to usability principles, and organized into 2 levels of hierarchy: generic (69 recommendations) and specific (105 recommendations).

**Conclusions:**

This study shows that user interface design recommendations can be divided according to usability principles and organized into levels of detail. Moreover, this study reveals that some recommendations, as they address different technologies and interaction paradigms, need further work.

## Introduction

In the context of digital solutions, user interface design consists of correctly defining the interface elements so that the tasks and interactions that users will perform are easy to understand [1]. Therefore, a good user interface design must allow users to easily interact with the digital solution to perform the necessary tasks in a natural way [2]. Considering that a digital solution is used by an individual with specific characteristics in a particular context [3-7], when developing a digital solution, designers must pay attention to a high number of components of user interface design, such as color [8] typography [1], navigation and search [9], input controls, and informational components [10].

Digital solutions and their interfaces must be accessible to all audiences and aimed at universal use in an era of increasingly heterogeneous users [3,4,11-17]. Therefore, designers should also be aware of broad and complex issues such as context-oriented design, user requirements, and adaptable and adaptive interactive behaviors [5]. The universal approach to user interface design follows heuristics and principles systematized by different authors over the years [18-20], but these are generic guidelines, and examples of how they can be operationalized in practice are scarce.

The literature presents many user interface design recommendations for varied digital solutions and users [21-25]. However, the absence of a detailed critical analysis that compares, evaluates, and validates existing recommendations is likely to facilitate an increasing number of similar recommendations [12,26-29]. Although existing recommendations refer to specific technologies, forms of interaction, or end users, the content of some recommendations is generic and applicable to varied technologies and users, such as “always create good contrast between text and page background” [30]; “color contrast of background and front content should be visible” [23]; “leave space between links and buttons” [30]; and “allow a reasonable spacing between buttons” [31]. These illustrative examples highlight the need to aggregate, analyze, and validate existing recommendations on user interface design. Accordingly, this study aimed to synthesize existing guidelines into a practical set of recommendations that could be used to guide user interface design for different technologies. This is important because it contributes to the standardization of good practices and will conceivably allow for better interface design achieved at earlier stages of product development.

## Methods

### Background

**Figure 1:**
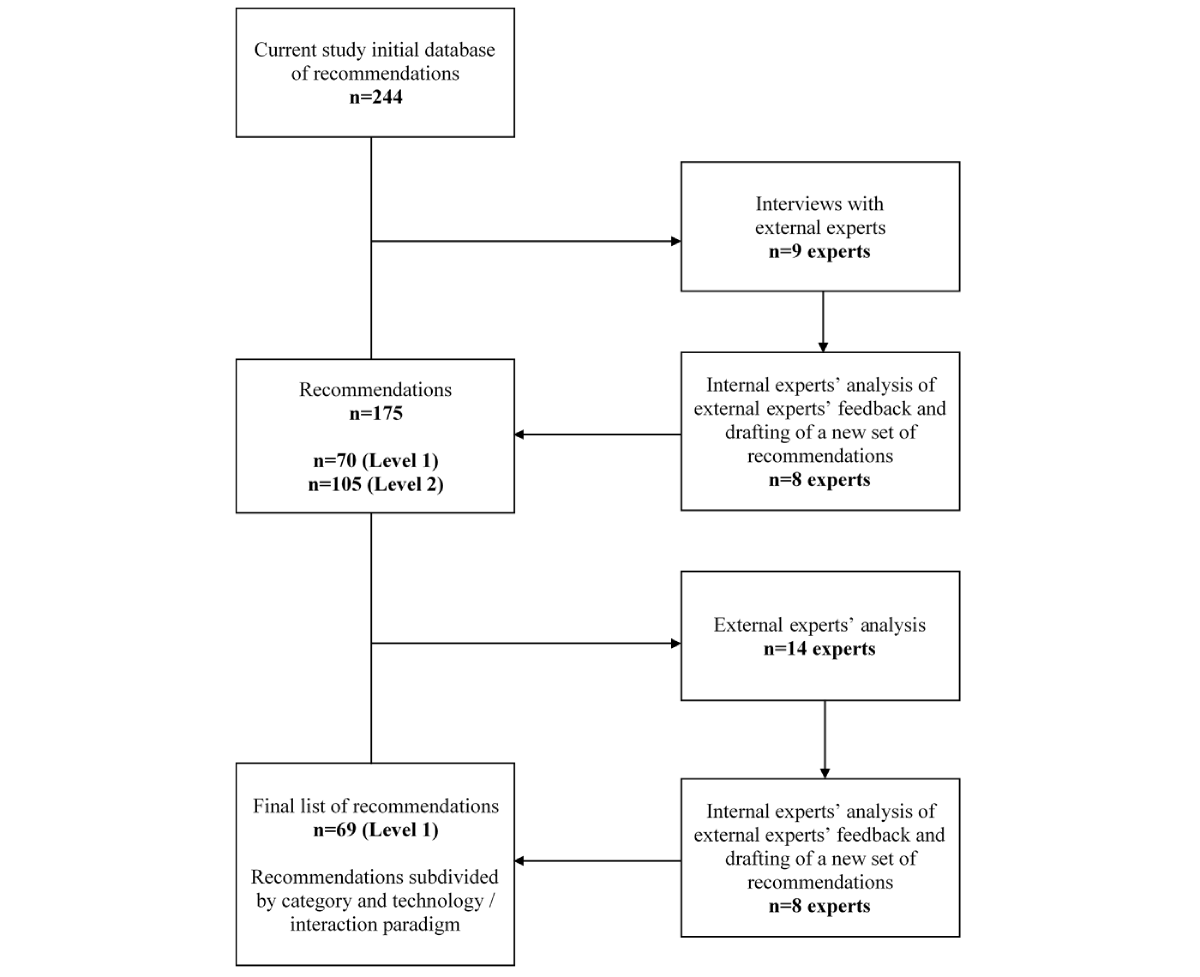
Steps of analysis of the user interface design recommendations.

In a previous work, 244 interface recommendations were identified, and they formed the basis for this study [32]. The identification of the 244 recommendations combined multiple sources: (1) our previous work [33], (2) a purposive search on Scopus database, and (3) inputs provided by experts in the field of interface design. The references identified through all 3 steps were extracted into an Excel (Microsoft) database with a total of 1210 recommendations. We screened this database and looked for duplicated recommendations. During this analysis, very generic recommendations were also deleted, and recommendations addressing similar content were merged. The resulting database, with 194 recommendations, was analyzed by 10 experts in user interface design recruited among SHAPES (Smart and Health Ageing through People Engaging in Supportive Systems) [34] project partners, who added another 62 recommendations, resulting in 256 recommendations. A further analysis identified 12 duplicated references that were deleted, resulting in a final list of 244 recommendations. The large number of recommendations was deemed impractical, and further action was necessary. Building on this prior research, a set of recommendations on user interface design were engendered following 4 steps: (1) interview with user interface design experts, (2) analysis of the experts’ feedback and drafting of a set of recommendations, (3) reanalysis of the shorter list of recommendations by a group of experts, and (4) refining and finalizing the list. Each of these steps is detailed below, and the whole process is illustrated in Figure 1.

### Interview With User Interface Design Experts

An Excel file with the 244 user interface design recommendations was sent to external experts in the field of user interface design. For an individual to be considered an expert, they had to meet at least 1 the following criteria: (1) have designed user interfaces for at least 2 projects/digital solutions or (2) have participated in the evaluation of user interfaces for at least 2 projects/digital products.

An invitation email was sent to experts explaining the objectives of the study, along with a supporting document with the 244 recommendations. They were asked to analyze the recommendations and report on (1) repetition/relevance, (2) wording, (3) organization, and (4) any other aspect they felt relevant. They were given approximately 4 weeks to analyze the 244 recommendations and send their written analysis and comments back to us. Subsequently, they were asked to attend a Zoom (Zoom Video Communications) meeting aimed at clarifying their views and discussing potential contradictory comments. The written comments (sent in advance by the experts) were used to prepare a PowerPoint (Microsoft) presentation where recommendations and respective comments (anonymized) from all experts were synthetized. This presentation was used to guide the Zoom meeting discussion. To maximize the efficiency of the discussion, recommendations without any comments and those that received similar comments from different experts were not included in the presentation. For recommendations with contradictory comments, the facilitator led a discussion and tried to reach a consensus. For recommendations with comments from a single expert, the facilitator asked for the opinion of other experts. The Zoom meeting was facilitated by one of the authors (AIM) and assisted by another (author CD) who took notes. The facilitator encouraged the discussion and exchange of opinions from all experts participating in each meeting. The Zoom meetings were recorded, and the experts’ arguments were transcribed and analyzed using content analysis by 2 authors (AIM and AGS) with experience in qualitative data analysis. Written comments sent by the experts as well as comments and relevant notes made during the meeting were transposed into a single file and subject to content analysis. After transcription, the notes were independently read by both the aforementioned authors and grouped into themes, categories, and subcategories with similar meaning [35]. Themes, categories, and subcategories were then compared, and a consensus was reached by discussion.

### Analyzing Experts’ Feedback and Drafting a Set of Recommendations

The authors of this manuscript (internal experts), including individuals with expertise on content analysis and on user interface design and usability, participated in a series of 6 meetings that were approximately 2 to 3 hours in duration each. These meetings, which took place between January and April 2021 and were held online, aimed to analyze the comments made by external experts in the previous step. Based on the experts’ comments, each recommendation was either (1) not changed (if no comments were made by the experts), (2) deleted, (3) merged with other complementary recommendations, (4) rewritten, or (5) broken up into more than 1 recommendation. The decisions were based on the number of experts making the same suggestion, alignment with existing guidelines, and coherence with previous decisions for similar recommendations. In addition, based on external experts’ suggestions, the recommendations were organized as follows: (1) hierarchical levels according to level of detail and interdependency, (2) usability principles, and (3) type of technology and interaction paradigm.

### Reanalyzing the Shorter List of Recommendations

To further validate decisions made by the internal panel and explore the possibility of reducing the number of recommendations, the set of recommendations resulting from the previous step (and its respective organization according to hierarchical levels and principles) was reanalyzed by an additional external panel of experts. Once again, to be considered an expert, individuals had to meet the previously identified criteria for experts (have designed user interfaces for at least 2 projects/digital products or have participated in the evaluation of user interfaces for at least 2 projects/digital products). An online individual interview was conducted in May 2021 with each expert by one of the authors (CD). Experts had to answer 3 questions about each of the recommendations: (1) Do you consider this recommendation useful? (Yes/No); (2) Do you consider this recommendation mandatory? (Yes/No); and (3) Do you have any observation/comment on any recommendations or on its organization? The first question was used to determine the inclusion or exclusion of recommendations, and the second one was used to inform on the priority of recommendations through the possibility of having 2 sets of recommendations: 1 mandatory and 1 optional. The third question aimed to elicit general comments on both individual recommendations and their organization. Consensus on the first 2 questions was defined as 70% or more of the experts signaling a recommendation with “Yes” and less than 15% of experts signaling the same recommendation with “No.” Qualitative data from the third question was independently analyzed by 2 authors (CD and AGS) using content analysis, as previously described.

### Refining and Finalizing the List of Recommendations

The internal panel of experts (the authors of this study) had an online meeting in which findings of the previous step were presented and discussed, and amendments to the existing list of recommendations were decided to generate the final list of user interface design recommendations.

### Ethical Considerations

This study focused on the analysis of previously published research and recommendations; therefore, ethical approval was considered unnecessary.

## Results

### Interview With User Interface Design Experts

A total of 9 experts participated in this step of the study: 5 females and 4 males with a mean age of 39.1 (SD 4.3) years. The participants were user interface designers (n=3, 33%) and user interface researchers (n=6, 67%) who had a background in design (n=6, 67%), communication and technology sciences (n=2, 22%), or computer engineering (n=1, 11%). A total of 3 meetings with 1 to 3 participants were conducted with a mean duration of 2 hours. Of the 244 recommendations, 166 (68%) were commented on by at least 1 expert.

Regarding the analysis of the interviews and written information sent by the experts, it was possible to aggregate commentaries into 2 main themes: (1) wording and content of recommendations and (2) organization of recommendations. The first theme was divided into 5 categories: (1) not changed (if no comments were made by the experts); (2) deletion of recommendations (because they were not useful or were contradictory); (3) merging of recommendations (to address complementary aspects of user interface design); (4) rewriting of recommendations (for clarity and coherence); and (5) splitting recommendations into more than 1 (because they included different aspects of user interface design). Of the 244 recommendations, external experts suggested that 108 should be merged (usually pairs of recommendations but could also include more than 2 recommendations), 29 should be rewritten, 4 should be split into more than 1, and 44 should be deleted. Among the recommendations, 78 received no comment. For 19 recommendations, it was not possible to reach consensus in the interview phase.

The second theme (organization of the recommendations) was divided into 2 categories: (1) hierarchization of recommendations and (2) grouping of recommendations. This last category was subdivided into 2 subcategories: (1) grouping of recommendations according to usability principles and (2) grouping of recommendations according to whether they apply to digital solutions in general or to specific digital solutions/interaction paradigms. Examples of quotations that support these categories and subcategories are presented in Table 1. Regarding the grouping of recommendations according to usability principles, the categories proposed by 5 experts (Table 1) were reorganized and merged into 12 categories: feedback, recognition, flexibility, customization, consistency, errors, help, accessibility, navigation, privacy, visual component, and emotional component. The mapping of the categories proposed by the experts and the 12 categories (named principles) are presented in Multimedia Appendix 1.

**Table 1:** Categories and subcategories of the theme “organization of recommendations,” quotations supporting the categories, and number of experts that made comments in each category/subcategory.

Categories	Subcategories	Citations (examples)	Experts, n (%)
Hierarchization	N/A^a^	There are recommendations with different levels of detail, and they are all placed at the same level; some recommendations correspond to guidelines, others are practical design indications. [E^b^6, male] It would be interesting to organize the recommendations based on the relationship between them. A high-level recommendation contains low level recommendations. [E8, male] It makes sense to split into layers. I suggest dividing them into recommendations applicable to all and into standards, with a very high level of detail (button size, space between buttons etc). [E1, female]	4 (44)
Grouping of recommendations	Design principles	During our analysis, we organized the requirements into categories to assist us. [E7, female] To be able to do the analysis, I had to code each recommendation to more easily identify the ones that could be grouped according to that category and thus detect repetitions and redundancies. [E6, male] To make it easier, I created categories according to Nielsen's 10 usability heuristics and 5 design principles. [E3, female] Of the 9 experts, 5 suggested categories for grouping recommendations: Feedback; users/confusion, errors; human-computer dialogue; system behavior; navigation; presentation; system; users; instruction/information; user control; personalization; system; screen reader; users/cognitive load; system/devices; instructions; user/emotions; design considerations; gamification; users/sensory. [E7, female] System status; feedback; task execution; navigation/ interaction; organization/structure of information; attention-orientation; information hierarchy; iconography; visual composition of information and of interaction elements; naming; navigation; accessibility; input device and interaction; attention-orientation; learnability; interface customization; complexity and density of information; typography/legibility and formatting; media controllers; color and contrast; navigation/task execution; information representation/mental models. [E6, male] Feedback; visibility; multimodality; help; recognition; mental burden; control; design; real world; language; flexibility; errors; consistency; accessibility; personalization; search history; animation; efficiency; number of steps; shortcuts; hierarchy; legibility; color; cultural context; security; body; discovery; emotion; gamification; predictive; privacy. [E3, female] *Feedback; text information; user profile; layout; navigation; content; tasks; errors; consistency; input; ergonomic; emotional; security; gamification*. [E2, female] Visual dimension; dimension of information architecture; dimension of social presence; interaction dimension and dimension of user experience. [E1, female]	7 (78)
	Generic vs specific to technology/ interaction paradigms	The recommendations focus on different types of interactive products (feet, audio interaction, robotics, etc). It would make sense in the end, too, to organize and separate the recommendations by product types. [E6, male] I noticed that some recommendations distinguish interaction modalities (with voice, gestures, use of the feet, etc). I selected those that were general and those that were specific for these groups. [E3, female] All recommendations were put in the same bag, regardless of the detail. I think we are using design principles, guidelines, and standards. Standards only make sense when applied to a specific system, and it is very difficult to classify them without having in mind the system being evaluated. Design standards are derived from design principles and guidelines but applied to specific products. What is being done here is very rich and interesting, but it can lead to a “Frankenstein product,” because the recommendations depend on several factors. I think that at this point you should work with principles, point out the guidelines and check the recommendations for the different products. [E1, female] As we are talking about recommendations that cut across different types of interactive products (feet, audio interaction, robotics, etc), it would make sense in the end, too, to organize and separate the recommendations by product types. [E6, male] They are related in the area of interaction, but each one talks about a different interface. [E3, female] Of the 9 experts, 3 suggested categories for grouping recommendations: Voice interaction; feet interaction; robot. [E7, female] Generic; generic/user centered. [E6, male] Voice; feet; real world; robot; touch; click; text; gestures. [E2, female]	3 (33)

^a^N/A: not applicable.

^b^E: expert.

### Analysis of Experts’ Feedback and Reanalysis of the Recommendations

Based on the external expert’s comments, the recommendations were reanalyzed. Of the 244 recommendations, 61 (25%) were deleted because they were duplicated or redundant, 48 (19.7%) were merged with other complementary recommendations, 62 (25.4%) were rewritten for clarification and language standardization, 14 (5.7%) were split in 2 or more recommendations, and 59 (24.2%) were not changed. This resulted in a preliminary list of 175 recommendations. Table 2 compares the external experts’ recommendations and internal experts’ final decision.

The 175 recommendations were then categorized into 12 mutually exclusive principles (feedback, recognition, flexibility, customization, consistency, errors, help, accessibility, navigation, privacy, visual, and emotional) and within each principle, organized into 2 levels of hierarchy according to the specificity/level of detail.

Of the 175 recommendations, 70 were categorized as level 1 and were generic recommendations applied to all digital solutions, and 105 recommendations were linked to 1 first level recommendation and subdivided by type of digital solution/interaction paradigm. The recommendations of both levels are linked, as level 2 recommendations detail how level 1 recommendations can be implemented. For example, the level 1 recommendation that “the system should be used efficiently and with a minimum of fatigue” is linked to a set of level 2 recommendations targeted at specific interaction paradigms, such as feet interaction and robotics: (1) “In feet interaction, the system should minimize repetitive actions and sustained effort, using reasonable operating forces and allowing the user to maintain a neutral body position,” and (2) “In robotics, the system should have an appropriate weight, allowing the person to move the robot easily (this can be achieved by using back drivable hardware).” Table 3 shows the distribution of the 175 recommendations.

**Table 2:** Comparison of external expert’s recommendations and internal experts’ decision.

Type of action	External experts’ recommendations (N=263)^a^, n (%)	Internal experts’ decision (N=244), n (%)
Deleted	44 (16.7)	61 (25)
Merged	108 (41.1)	48 (19.7)
Rewritten	29 (11)	62 (25.4)
Split	4 (1.5)	14 (5.7)
Not changed	78 (29.7)	59 (24.2)

^a^Consensus was not possible for 19 recommendations.

**Table 3:** Distribution of recommendations by level and category.

Category	Level 1, (N=70), n	Level 2, (N=105), n	Technology/interaction paradigm	Total (N=175), n
Feedback	6	5	Feet Interaction: 1 Robotics: 1 Voice Interaction: 2 Web/Mobile: 1	11
Recognition	5	12	Feet interaction: 1 Robotics: 1 Voice interaction: 5 Web/mobile: 5	17
Flexibility	6	10	Feet interaction: 1 Robotics: 4 Voice interaction: 2 Web/mobile: 3	16
Customization	7	6	Feet interaction: 1 Robotics: 1 Voice interaction: 3 Web/mobile: 1	13
Consistency	2	2	Voice interaction: 2	4
Errors	5	7	Feet interaction: 3 Robotics: 1 Voice interaction: 3	12
Help	3	2	Robotics: 1 Web/mobile: 1	5
Accessibility	8	23	Feet interaction: 8 Robotics: 4 Web/mobile: 11	31
Navigation	6	6	Feet interaction: 3 Web/mobile: 3	12
Privacy	3	5	Digital solutions: 5	8
Visual component	16	22	Feet interaction: 5 Robotics: 2 Web/mobile: 15	38
Emotional component	3	5	Feet interaction: 1 Robotics: 3 Digital solutions: 1	8

### Reanalysis of the Shorter List of Recommendations by Experts

A total of 14 experts (8 females and 6 males) with a mean age of 35 (SD 8.8) years old provided feedback on recommendations. Experts were user interface designers (n=6, 43%) and user interface researchers (n=8, 57%) who had a background in design (n=8, 57%) or communication and technology sciences (n=6, 43%). The interviews lasted up to 2 hours each.

All the 175 recommendations reached consensus for the usefulness question. However, for question 2 (Do you consider this recommendation mandatory?), there was consensus that 54 (77%) level 1 recommendations were mandatory. The remaining 16 (23%) level 1 recommendations were considered by 5 (36%) to 9 (64%) experts as not mandatory. For the 105 level 2 recommendations, there was consensus that 91 (87%) were mandatory, and the remaining 14 were not considered mandatory by 5 (36%) to 9 (64%) experts.

Experts’ comments were aggregated into 5 main themes: (1) deletion or recategorizing of recommendations, (2) consistency, (3) contradiction, (4) asymmetry, and (5) uncertainty. It was suggested that 1 recommendation be deleted (“The system should be free from errors”), and another moved from the visual component category to the emotional component category. No other suggestions were made regarding the structure of the recommendations. There were comments related to the consistency, particularly regarding the need to use either British or American spelling throughout all recommendations and to consistently refer to “users” instead of “persons” or “individuals.” The remaining comments applied mostly to level 2 recommendations, for which experts identified contradictory recommendations (eg, accessibility: “In robotics, the system should meet the person’s needs, be slow, safe and reliable, small, easy to use, and have an appearance not too human-like, not patronizing or stigmatizing*”* vs emotional: “In robotics, the system should indicate aliveness by showing some autonomous behavior, facial expressions, hand/head gestures to motivate engagement, as well as changing vocal patterns and pace to show different emotions”). Experts also commented on the asymmetry across the number of level 2 recommendations linked to level 1 recommendations and on the asymmetry regarding the number of recommendations per type of technology and interaction paradigm. In addition, experts were uncertain about the accuracy of the measures indicated in the recommendations (eg, visual: “In robotics, the system graphical user interface and button elements should be sufficiently big in size, so they can be easily seen and used, about ~20 mm in case of touch screen, buttons” vs visual: “In feet interaction, the system should consider an appropriate interaction radius of 20 cm for tap, 30 cm at the front, and 25 cm at the back for kick”).

### Refining and Finalizing the List of Recommendations

Based on the experts’ comments and issues raised in the previous step, the term “users” was adopted throughout the recommendations, 1 recommendation was removed, and 1 was moved from the visual component to the emotional component. In addition, all level 1 recommendations for which no consensus was reached on whether they were mandatory were considered not mandatory (identified by using the word “may” in the recommendation). The internal panel also recognized that level 2 recommendations cannot be used to guide user interface design in their current stage and that further work is needed. Therefore, a final list of 69 generic recommendations is proposed (Multimedia Appendix 2).

## Discussion

### Principal Findings

To the best of our knowledge, this is the first study that attempted to analyze and synthetize existing recommendations on user interface design. This was a complex task that generated a high number of interdependent recommendations that could be organized into hierarchical levels of interdependency and grouped according to usability principles. Level 1 recommendations are generic and can be used to inform the user interface design of different types of technology and interaction paradigms. Meanwhile, level 2 recommendations are more specific and therefore apply to different types of technology and interaction paradigms. Furthermore, the level of detail and absence of evidence that they had been validated raised doubts about their validity.

The external experts’ suggestions formed the basis for the internal experts’ (our) analysis. However, there is a discrepancy between the analysis of both panels of experts in terms of the number of recommendations that should be deleted, merged, rewritten, fragmented, or not changed. This was because when analyzing the recommendations, the internal panel verified that there were more recommendations to delete that were repeated or generic beyond those already identified by the external panel. It is likely that these were missed due to the high number of recommendations, which made the analysis a time-consuming and complex task. Furthermore, changing 1 recommendation in line with external experts’ suggestions resulted in subsequently having to change other recommendations for coherence and consistency, resulting in a higher number of recommendations that were rewritten. In addition, there was a lack of consensus among external experts, leaving the final decision to the internal experts (us), further contributing to discrepancies.

Regarding the organization of the recommendations, the division into 2 hierarchical levels based on the specificity/level of detail resulted from the external experts’ feedback and aimed at making the consultation of the list of recommendations easier. This type of hierarchization in levels of detail was also used in previous studies aimed at synthetizing existing guidelines [23,36].

The recommendations were grouped into 12 categories, which closely relate to existing usability principles (feedback, recognition, flexibility, customization, consistency, errors, help, accessibility, navigation, and privacy [18,37-39]). Usability principles are defined as broad “rules of thumb” or design guidelines that describe features of the systems to guide the design of digital solutions [18,40]. Additionally, they are oriented to improve user interaction [3] and impact the quality of the digital solution interface [41]. Therefore, having the recommendations organized in a way that maps these principles helps facilitate a practical use of the recommendations proposed herein, as these usability principles are familiar to designers and are well established, well known, and accepted in the literature [23,42].

The results showed an asymmetry in the number of recommendations categorized into each of the 12 usability principles (eg, for level 1, consistency has 2 recommendations while the visual component has 16 recommendations). This discrepancy suggests that some areas of user interface design such as the visual component might be better detailed, more complex, or more valued in the literature, but can also suggest that the initial search might not have been comprehensive enough, as it included a reduced number of databases [32]. Nevertheless, the heterogeneity between categories does not influence its relevance, as it is the set of recommendations as a whole that influences the user design interface of a digital solution.

The number of level 2 recommendations aggregated under each level 1 recommendation is also uneven. Most of the level 2 recommendations that resulted from this study concern web and mobile technologies because their utilization is widespread among the population [43] and therefore more likely to have design recommendations reported in the literature [23,31,44,45]. On the other hand, emerging technologies like robotics and interaction paradigms (eg, gestures, voice, and feet) represent new challenges for researchers, and recommendations are still being formulated, resulting in a lower number of specific recommendations that are published [46-49]. Moreover, the level 2 recommendations raised doubts among experts, namely regarding (1) the lack of consensus on whether they were mandatory or not, (2) apparent contradictions between recommendations, and (3) uncertainty regarding the accuracy of some recommendations, particularly those very specific (eg, the recommendations on the size of the buttons in millimeters). These aspects suggest that level 2 recommendations need further validation in future studies. No data was found on how the authors of the recommendations arrived at this level of detail and how the exact recommendation might vary depending on the target users [50,51], the type of technology [49], interaction paradigm [46], and the context of use [52]. Validation of the level 2 recommendations might be performed by gathering expert’s consensus on the adequacy of recommendations by type of technology/interaction paradigm and involving real users to test if the specific user interfaces that fulfill the recommendations improve usability and user experience [50,53].

We believe that level 1 recommendations apply to different users, contexts, and technologies/interaction paradigms and that the necessary level of specificity will be given by level 2 recommendations, which can be further operationalized into more detailed recommendations (eg, creating level 3 recommendations under level 2 recommendations). For example, recommendation 1 from the recognition category states that “the system should consider the context of use, using phrases, words, and concepts that are familiar to the users and grounded in real conventions, delivering an experience that matches the system and the real world,” which is an example of applicability to different contexts such as health or education. Similarly, recommendation 1 from the flexibility category states that “the system should support both inexperienced and experienced users, be easy to learn, and to remember, even after an inactive period,” also showing adaptability to different types of users. Nevertheless, the level of importance of each level 1 recommendation might vary. For example, recommendation 6 of the flexibility category, which states that “the system may make users feel confident to operate and take appropriate action if something unexpected happens,” was not considered mandatory by the panel of external experts. However, one might argue that it should be considered mandatory in the field of health, where the feeling of being in control and acting immediately if something unexpected happens is of utmost importance. Therefore, both level 1 and level 2 recommendations require further validation across different types of technology and interaction paradigms but also for different target users and contexts of use. Also required are investigations to determine whether their use results in better digital solutions, and particularly for the health care field, increases adhesion to and effectiveness of interventions.

In synthesis, although this study constitutes an attempt toward a more standardized approach in the field of user interface design, the set of recommendations presented herein should not be seen as a final set but rather as guides that should be critically appraised by designers according to the context, type of technology, type of interaction, and the end users for whom the digital solution is intended.

### Strengths and Limitations

The strengths of this proposed set of recommendations are that it was developed based on multiple sources and multiple rounds of experts’ feedback. However, although several experts were involved in different steps of the study, it cannot be guaranteed that the views of the included experts are representative of the views of a broader community of user interface design experts. Another limitation of this study is that the initial search for recommendations might not have been comprehensive enough. Nevertheless, external experts were given the possibility of adding recommendations to the list, and none suggested the need to include additional recommendations. The list of level 2 recommendations is a work in progress that should be further discussed and changed considering the technology/paradigm of interaction. Finally, some types of technologies and interaction paradigms are not represented in the recommendations (eg, virtual reality), and it would be important to have specific recommendations for all types of technologies and interaction paradigms in the future.

## Appendix

Multimedia Appendix 1 Mapping of the categories proposed by the experts and the final categories.

Multimedia Appendix 2 Final list of 69 generic recommendations proposed.

## Abbreviations

SHAPES: Smart and Health Ageing through People Engaging in Supportive Systems
