# Supplementary material for: Defining Recommendations to Guide User Interface Design: Multimethod Approach
Source: JMIR Hum Factors. 2022 Sep 30;9(3):e37894. doi: 10.2196/37894 (PMC9568819; doi:10.2196/37894)
Supplement: Multimedia Appendix 1 [file humanfactors_v9i3e37894_app1.docx]

Multimedia Appendix 1. Mapping of the categories proposed by the experts and the final categories.

| Categories | E7 | E6 | E3 | E2 | E1 |
| --- | --- | --- | --- | --- | --- |
| Feedback | Feedback;  Human-Computer dialogue;  System behavior;  Instruction/  information;  Users/  Sensory. | Feedback;  System status. | Feedback;  Visibility;  Efficiency;  Number of Steps. | Feedback;  Input. | Interaction dimension. |
| Recognition | Users/ cognitive load. | Attention-orientation;  Iconography;  Naming;  Learnability. | Recognition;  Mental burden;  Real World;  Language;  Search history;  Cultural context;  Discovery;  Predictive. | User profile |  |
| Navigation | Navigation;  System behavior. | Navigation/ interaction;  Media controllers;  Attention-orientation;  Information hierarchy. | Visibility;  Control;  Efficiency;  Hierarchy;  Shortcuts;  Number of Steps;  Discovery. | Navigation. | Dimension of Information Architecture. |
| Customization | Personalization;  User control;  Users/ cognitive load;  Users/sensory. | Interface customization. | Personalization;  Mental burden;  Language. | User profile. |  |
| Consistency |  | Organization/ Structure of Information;  Learnability. | Consistency;  Predictive. | Consistency;  Content;  Input. |  |
| Errors | Users confusion / errors. | Complexity and density of information;  Task execution. | Errors;  Efficiency;  Predictive. | Errors;  Tasks. |  |
| Help | Instruction/ information. | Complexity and density of information. | Help. |  |  |
| Accessibility | User control;  Screen reader. | Accessibility;  Learnability. | Accessibility;  Multimodality. | Ergonomy. |  |
| Flexibility | User control;  Personalization;  Users/ cognitive load. | Interface customization. | Flexibility;  Multimodality;  Mental burden;  Personalization. |  |  |
| Privacy |  |  | Security;  Privacy. | Security. |  |
| Visual | Design considerations;  Presentation. | Visual composition of information and of interaction element;  Color and contrast;  Typography/ legibility and formatting;  Information hierarchy;  Organization/  Structure of Information;  Iconography. | Design;  Color;  Legibility;  Hierarchy;  Animation. | Layout;  Content. | Visual Dimension;  Dimension of Information Architecture. |
| Emotional | User/ emotions;  Gamification. |  | Emotion;  Gamification. | Emotional;  Gamification;  User profile. | Dimension of Social Presence;  Dimension of User Experience. |
