# Supplementary material for: Defining Recommendations to Guide User Interface Design: Multimethod Approach
Source: JMIR Hum Factors. 2022 Sep 30;9(3):e37894. doi: 10.2196/37894 (PMC9568819; doi:10.2196/37894)
Supplement: Multimedia Appendix 2 [file humanfactors_v9i3e37894_app2.docx]

**Multimedia Appendix 2. Final list of 69 generic recommendations proposed.**

| Feedback | 1. The system should keep users informed about the system's performance and the result of its actions. Feedback should be simple, standardized and include enough information, and if necessary, consider, complementary forms of feedback (e.g., audio and/or video tutorials). |
| --- | --- |
|  | 2. The system instructions should be short, simple, appropriate, understandable, positive and the message should be discrete, visible and/or easily retrievable. |
|  | 3. The system error messages should be simple and positive, accurately indicate the problem and constructively suggest an easy-to-follow solution. |
|  | 4. The system should inform users about updates or features/services adds, in order to avoid user interface updates and adaptations that profoundly alter the system's behavior. |
|  | 5. The system may provide an integrated presentation of the information retrieved from multiple sensors and present it in a clear format. |
|  | 6. The system may include visual, auditory, or tactile signals that give distinctive and informative feedback to manage users' attention to what is really important at each precise moment. |
| Recognition | 1. The system should consider the context of use, using phrases, words and concepts that are familiar to the users, grounded in real conventions, delivering an experience that matches the system and the real world, avoiding the users' cognitive load and predicting learning mechanisms to support the users' interaction. |
|  | 2. The system should match the most relevant social norms, ensuring that the experience is delivered according to what the users expect and to their cultural and social context. |
|  | 3. The system should allow users to easily recognize user functions and system options through interaction, affordances, and visible features, save recent actions/interactions and allow recovering short-term memory. |
|  | 4. The system information should be simple, concise, and to the point. |
|  | 5. The system elements should use natural cues, efficient interaction language, material objects similar to real-world objects, and be based on the user’s native language. |
| Flexibility | 1. The system should support both inexperienced and experienced users, be easy to learn, and to remember, even after an inactive period. |
|  | 2. The system should offer different options for user control, allowing alternative and flexible flows of interaction, such as including functions that allow the user to easily leave an unwanted state or interaction (e.g., provide emergency exits and support to easily undo and redo an action), and functions that support the users’ freedom (e.g., ability to rewind or advance screens, pause, resume, restart, or end activities). |
|  | 3. The system should be designed based on the principle of flexibility, offering different ways to perform the same task (e.g., through shortcut keys and different menu options). |
|  | 4. The system may fit individual tasks within whatever modality is being used – auditory, visual, or motor/kinesthetic –, accommodating a wide range of individual preferences and capabilities. |
|  | 5. The system may provide compatibility with a variety of techniques or devices (e.g., assistive technologies or assistive devices). |
|  | 6. The system may make users feel confident to operate and take appropriate action if something unexpected happens. The system may automatically predict mode changes (manual/automatic control) through simple gestures or actions. |
| Customization | 1. The system should offer easy ways for the user to change the text size on the screen. |
|  | 2. The system may provide different ways to login (e.g., PIN number, face recognition or fingerprint). |
|  | 3. The system may have personalized style guides, allowing users to choose themes, font sizes, colors, and the activation of specific and personalized options. |
|  | 4. The system may allow users to customize specific functions, based on their differing needs, namely tailoring frequent actions, and ignoring or dismissing undesirable features. |
|  | 5. The system may allow users to customize the experience, providing the possibility to control the amount of information displayed and how it is stored (e.g., details provided in the feedback). |
|  | 6. The system may learn from user interaction to personalize their experience and retrieve granular feedback during the interaction. |
|  | 7. The system may provide adaptability according to the users’ learning pace. |
| Consistency | 1. The system should ensure consistency in appearance, workflow, functionality, feedback, and terminology to facilitate the users’ next interaction. |
|  | 2. The system screen layout, navigation and terminology should be simple, clear, and consistent. |
| Errors | 1. The system should provide users with mechanisms to prevent, minimize, detect, correct, and recover from errors, as well as warnings about hazards and errors, ensuring fail-safe features. |
|  | 2. The system should ensure that important or frequently needed functions can be easily accessed and understood by users, to avoid inappropriate choices or errors. The system should also provide the confirmation option before committing the user to any critical action (e.g., exclusion). |
|  | 3. The system should minimize the users’ short-term memory load and support recognition instead of remembering through visible and easily located objects, actions, and options, and provide contextualized, relevant, appropriate, and useful information for the users' tasks. |
|  | 4. The system should ensure focus on one task at a time and clearly indicate the name and status of the task at all times. |
| Help | 1. The system should provide easily located help, specific to the task in question and written, in order to guide the user to the solution through the necessary steps. |
|  | 2. The system should support efficient evocation, enabling its functionalities and services whenever users request it, and provide help and additional information to assist the users during the interaction. |
|  | 3. The system may offer the user opportunities to practice the actions. |
| Accessibility | 1. The system should be free of stereotypes and social biases. |
|  | 2. The system should be capable of effectively providing necessary information to the user, regardless of ambient conditions. |
|  | 3. The system should not overload the users’ cognitive, visual, auditory, tactile, or motor limits. |
|  | 4. The system should be used efficiently and with a minimum of fatigue. |
|  | 5. The system should provide alternative content, ensuring the accessible automatically specified content and helping users to manage it for non-text content, preserving the accessibility of the system information. |
|  | 6. The system may have an appropriate interface to the target user and be adaptable according to the users physical and cognitive abilities, as well as their level of knowledge in technological interfaces. |
|  | 7. The system may provide ample time to read information, avoiding the use of interaction timeouts. |
|  | 8. The system may provide appropriate size and space for approach, reach, manipulation, and use, regardless of the users’ anthropometric characteristics. |
| Navigation | 1. The system should provide the options and information in a logical sequence. |
|  | 2. The system should provide easily located and unambiguous means of navigating to other content. |
|  | 3. The system should ensure that the "Back" button behaves predictably. |
|  | 4. The system should ease complex tasks using progressive disclosure, indicating preferred actions or next steps, or locating controls near the objects that users want to manage. |
|  | 5. The system may have a small number of steps of user-system dialog to ensure a greater perceived efficiency of the interaction with the system. |
|  | 6. The system may offer enhanced navigation by including a search engine and providing content text-search, catering for spelling errors. |
| Privacy | 1. The system should guarantee security and privacy as the default setting and provide a clear and easy to understand privacy policy. |
|  | 2. The system should ensure safety for all users. |
|  | 3. The system should provide alternatives to the needs of different users, namely with regard to privacy, security and protection, which should be equally available to everyone. |
| Visual | 1. The system should keep the clutter to a minimum. |
|  | 2. The system may concentrate the information mainly in the center of the screen. |
|  | 3. The system should avoid deep hierarchy, and visually group information into meaningful categories. |
|  | 4. The system should provide adequate contrast between essential information and its surroundings, favoring the readability of essential information. |
|  | 5. The system elements should be organized consistently and with spacing between them. |
|  | 6. The system should provide simple, meaningful, clear, easy to understand and labeled icons, ensuring that there are visual cues for the user. |
|  | 7. The system should ensure that the buttons and icons increase as the text size increases. It should also ensure that there is enough space between the buttons to prevent multiple elements from being triggered incorrectly. |
|  | 8. The system should have buttons large enough. |
|  | 9. The system should include buttons and other actionable elements that should be surrounded by reasonable inactive space. |
|  | 10. The system interface may have a minimalist aesthetic, but it should be simple and clear. |
|  | 11. The system may avoid using multiple windows by providing the most relevant information in the main viewing window. |
|  | 12. The system information may be organized and presented according to its importance, ideally with an index, navigation icons and navigation trails, favoring a complete navigation experience. |
|  | 13. The system may group related objects and functions by user task or work activity. |
|  | 14. The system may highlight the important information and centralize it in the system's path for screen readers to find it. |
|  | 15. The system may maintain a typography and font size pattern for the interface. |
| Emotional | 1. The system should enable users to feel connected and have a sense of belonging. |
|  | 2. The system should enable the user to feel capable, competent, and effective. |
|  | 3. The system should provide joy and comfort to the users. |
|  | 4. The system may consider visual elements (e.g., color, typography, layout, images, graphics, personified icons) to trigger and retain positive emotional responses from the user. |
